# CUTANEOUS ZYGOMYCOSIS DUE TO *SAKSENAEA VASIFORMIS* IN AN IMMUNOCOMPETENT HOST

**DOI:** 10.4103/0019-5154.57621

**Published:** 2009

**Authors:** V P Baradkar, S Kumar

**Affiliations:** *From the Department of Microbiology, Lokmanya Tilak Municipal Medical College & General Hospital, Sion, Mumbai - 400 002, India.*

**Keywords:** *Cutaneous zygomycosis*, *immunocompetent host*, *Saksenaea vasiformis*

## Abstract

*Saksenaea vasiformis* is an emerging zygomycete species, most often associated with cutaneous, subcutaneous and rhino- orbito-cerebral infections. Herein, we report a case of cutaneous zygomycosis of face caused by *Saksenaea vasiformis* in a 54-year-old immunocompetent female. The diagnosis was carried out by microscopy using KOH mount, Gram staining, Gomori's methenamine silver staining, hemotoxylin and eosin staining and culture on Sabouraud's Dextrose agar without actidione. Slide cultures were put up on Czapek Dox agar, which showed typical flask-shaped sporangium with rhizoids. The patient was treated successfully with intravenous amphotericin B.

## Introduction

Zygomycetes are fungi that usually cause infections in immunocompromised patients. Zygomycetes contain two orders that contain genera and species of medical importance- *Mucorales* and the *Entomophthorales.* In general, the fungi of the order *Mucorales* cause more severe forms of the disease.[1] Most frequently encountered agents of human zygomycosis is *Rhizopus arrhizus,* but many other agents of the genera *Cunninghamellaceae, Mortierellaceae, Saksenaeceae* are also occasionally reported. Since 1980s, nosocomial zygomycosis manifesting as primary skin and wound infections caused by *Rhizopus, Absidia, Cunninghamella, Saksenaea vasiformis,* and *Apophysomyces elegans* are often being reported.[1,2]

Zygomycosis may manifest as rhinocerebral, pulmonary, abdominal, pelvic, cuatneous, and disseminated forms. *Saksenaea vasiformis* is the only known species of the genus *Saksenaeceae,* which was desribed by Saksenaea in 1953, based upon isolates from soil in India.[3] It has been isolated in soil samples from other geographic areas. The first human infections due to *Saksenaea vasiformis* were described by Ajello *et al.* in 1976.[4] *Saksenaea vasiformis* is most often associated with cuataneous or subcuataneous lesions after trauma. In this report, we describe a case of necrotising fascitis caused by *Saksenaea vasiformis* in an immunocompetent individual, which responded well to antifungal treatment.

## Case Report

**Figure 1:**
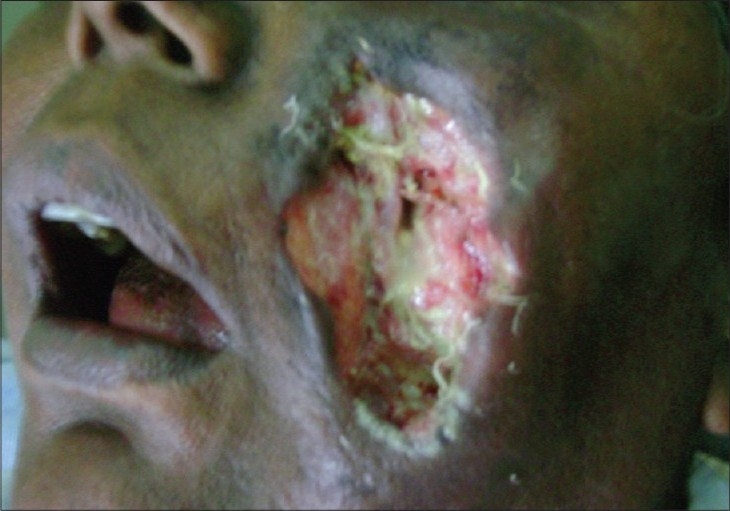
Ulcer on the face caused by *Saksenaea vasiformis*

**Figure 2:**
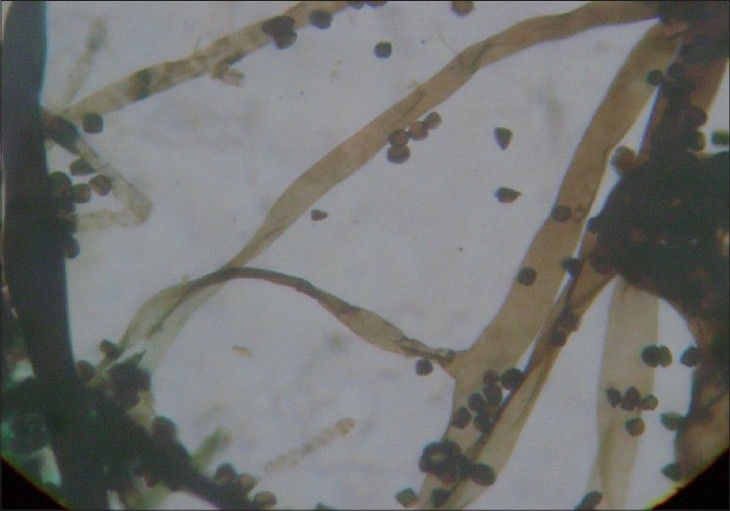
Aseptate hyphae in biopsy sample stained with GMS stain ×1000

A 54-year-old female presented with ulcerative wound over left cheek of approximately 6 × 6 cm in size for one month [Figure 1]. The patient gave a history of a small nodule three months back, which gradually increased in size before rupturing, following which, there was a whitish purulent discharge from the site. The patient did not give any history of any trauma to the affected part. The patient was nondiabetic, HIV seronegative and there was no history of any major illness in the past or any history suggestive of any immunocompromised state. Examination revealed that the patient was afebrile. The ulcer was slightly tender. Her blood investigations and liver and renal function tests were within normal limits. An emergency slough debridement was done and samples of excised skin and subcutaneous tissue were obtained for bacteriological and fungal culture and for histopathological examination. The material was processed for potassium hydroxide (KOH) preparation, Gram staining, Gomori's methenamine silver nitrate staining (GMS), hematoxylin and eosin staining. Gram stained smear showed broad aseptate hyphae, GMS staining showed brownish asepate hyphae [Figure 2], H and E stain also revealed aseptate hyphae. Bacterial cultures yield no growth of bacteria. There was creamish white cottony growth on Blood agar. There was no growth on Sabouraud's Dextrose (SDA) with actidione, however SDA without actidione kept at 22°C showed luxurious cottony growth after 48 h of incubation.

**Figure 3:**
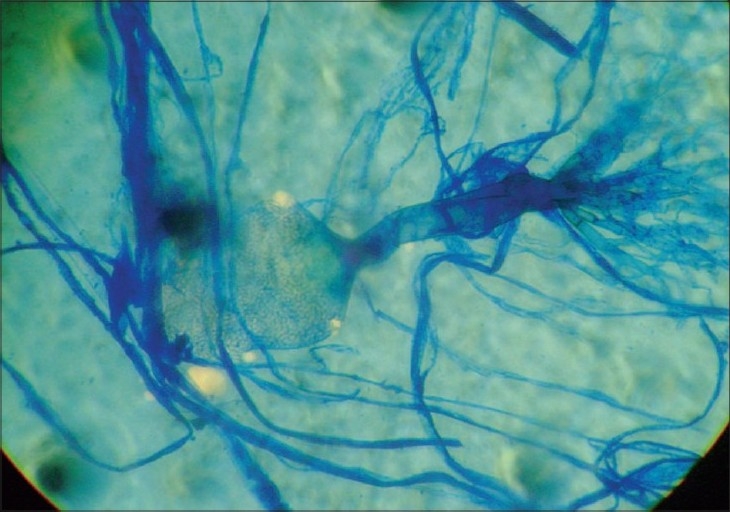
LPCB preparation from Czapek Dox agar showing typical flask shaped sporangium and rhizoids characteristic of *Saksenaea vasiformis*

Lactophenol cotton blue (LPCB) preparation from the growth showed only broad aseptate hyphae. No sporulation could be observed. Slide cultures were performed on Corn Meal agar, Czapek Dox agar and were incubated. Sporulation was only observed on Czapek Dox agar showed typical flask shaped sporangia and rhizoids [Figure 3]. Meanwhile, intravenous amphotericin B was started after the initial primary smear reports. The patient responded well to the treatment.

## Discussion

Zygomycosis (mucormycosis) is usually an acute fulminant infection of the immunocompromised host.[1] Cutaneous infections account for 16% of all forms of zygomycosis, with an associated mortality of 16%, compared to 67% for rhinocerebral, 83% for pulmonary, and 100% for disseminated infection. Most common agent is *Rhizopus species,* although others such as *Absidia, Mucor, Rhizomucor* are also frequently seen, whereas *Saksenaea vasiformis* and *Apophysomyces elegans* are rare pathogens.[1,2] Cutaneous and subcutaneous zygomycosis is less likely to be associated with severe systemic illness. Compared to other forms, where local predisposing factors such as burn, trauma, surgery, needle sticks and others play a major role,[1,2] most of the reported infections caused by *Saksenaea vasiformis* are subcutaneous.

Only two cases have been disseminated and one rhinocerebral infection has been reported.[5] Cutaneous infections may be primary or secondary, representing hematogenous dissemination from some other primary site. In primary disease, the infection may occur at the site of barrier break as in surgery or an indwelling catheter. Fatal disseminated infection with *Saksenaea vasiformis* in an immunocompromised woman has been reported by Torell *et al*.[7] Subcutaneous infections have been reported in a three-month-old infant and an eleven-year-old thalassemic child, who were successfully treated cases of tissue invasion following traumatic injury.[5]

The first case of subcutaneous zygomycosis by *Saksenaea vasiformis* in India was reported by Padhye *et al.*[8] in a rice mill worker in 1988. The infection involved the foot with multiple sinuses. Amputataion of the fore part of the foot followed by a split thickness graft and treatment with potassium iodide cured the infection. Primary cutaneous zygomycosis due to *Saksenaea vasiformis* has subsequently been reported from Chandigarh in 1977[9] and recently in 2006 by Padmaja *et al*.[10] from Vishakhapatnam.

*Saksenaea vasiformis* usually grows easily on routine mycological media, but it fails to sporulate on those media. It usually grows and produces characteristic flask-shaped sporangia when it is grown on nutritionally deficient medium such as agar blocks containing hyphal growth on sterile distilled water with the addition of sterilized yeast extract, 1-1.5% agar in saline or Czapek Dox agar. In most reported cases, sporulation was successfully induced using sterile distilled water method, although it may fail occasionally.[11] In the present case, sporulation was induced on Czapek Dox agar after 2 weeks of incubation.

In an immunocompetent individual, the infection usually remains localized and responds well to local debridement and antifungal therapy as happened in the present case.

In conclusion, *Saksenaea vasiformis* is increasingly being reported as a cause of subcutaneous zygomycosis. The pattern of infection is variable from mild infection to acute fatal infection, *Saksenaea vasiformis* has been reported to have favorable outcome after treatment in most of the cases, if early diagnosis is made and treatment is started earlier as reported by Padmaja *et al.* Hence, when a zygomycete species which fails to sporulate on routine media is isolated, the isolate should be cultured on nutritionally deficient media to induce sporulation so as to enable quick identification and to start treatment properly.
